# Comment on “Molecular Characterization of Animal *Fasciola* spp. Isolates from Kermanshah, Western Iran”

**Published:** 2018-08

**Authors:** Do Gyun LEE

**Affiliations:** Dept. of Environmental Engineering, Incheon National University, Incheon 22012, South Korea

## Dear Editor-in-Chief

In the previous work by Bozorgomid et al. (1), helpful information regarding the molecular epidemiology of fascioliasis in Kermanshah was demonstrated by characterizing the population genetic variability of *Fasciola* species. Although we think this study is very informative to researchers interested in the molecular epidemiology, the study contains Materials and Methods which are disputable.

The verification of the sequences of *Fasciola* species obtained in Bozorgomid et al. (1) has not been included in Materials and Methods section. Thus, the results of chimera detection should be provided as a further verification step since the sequences with undetected chimeras may be misinterpreted as novel species (2, 3). If chimeric DNA sequences are observed, they should be discarded for the further data analysis.

## References

[1] Bozorgomid A Nazari N Rahimi H (2016). Molecular Characterization of Animal *Fasciola* spp. Isolates from Kermanshah, Western Iran. Iran J Public Health, 45:1315–1321. 27957438 PMC5149495

[2] Edgar RC Haas BJ Clemente JC (2011). UCHIME improves sensitivity and speed of chimera detection. Bioinformatics, 27:2194–2200. 21700674 10.1093/bioinformatics/btr381 PMC3150044

[3] Acinas SG Sarma-Rupavtarm R Klepac-Ceraj V Polz MF (2005). PCR-induced sequence artifacts and bias: insights from comparison of two 16S rRNA clone libraries constructed from the same sample. Appl Environ Microbiol, 71:8966–8969. 16332901 10.1128/AEM.71.12.8966-8969.2005 PMC1317340

